# *Giardia duodenalis* Styles, 1902 Prevalence in Cattle (*Bos taurus* Linnaeus, 1758) in Europe: A Systematic Review

**DOI:** 10.3390/microorganisms11020309

**Published:** 2023-01-24

**Authors:** Maira Mateusa, Zanda Ozoliņa, Margarita Terentjeva, Gunita Deksne

**Affiliations:** 1 Institute of Food Safety, Animal Health and Environment BIOR, 1076 Riga, Latvia; 2 Faculty of Veterinary Medicine, Latvia University of Life Sciences and Technologies, 3001 Jelgava, Latvia; 3 Faculty of Biology, University of Latvia, 1004 Riga, Latvia

**Keywords:** giardiasis, assemblage, calves, risk factors, zoonosis, detection methods, public health

## Abstract

*Giardia duodenalis* Styles, 1902 is an infectious agent which can cause enteritic disease in cattle (*Bos taurus* Linnaeus, 1758) worldwide. As a zoonotic protozoan, it is important to acknowledge *Giardia* prevalence and assemblages found in cattle and risk factors associated with the *Giardia* infection in herds. This systematic review aims to estimate the prevalence of *G. duodenalis* and its assemblages in cattle and to identify the risk factors associated with *Giardia* infection in cattle in Europe. A systematic review was performed according to Preferred Reporting Items for Systematic reviews and Meta-Analyses (PRISMA) guidelines to collect information from published studies in Europe. A total of 1414 studies were identified and 17 relevant studies were included in this review. Mean *Giardia* prevalence in cattle in Europe was 35.1%, with the highest prevalence found in neonatal animals (39.6%), but mean heard prevalence was 67.0%. Mixed infections of *Giardia* assemblages A and E were found most frequently (55.6%), while assemblages A and B were found more frequently in animals under 24 months old. Risk factors, such as deep litter with run-out, prolonged calf contact with the dam, and seasonality, such as winter and spring, were found to be potential risk factors for *Giardia* presence in the herds.

## 1. Introduction

The genus *Giardia* consists of eight species—*G. agilis* Künstler, 1882, *G. ardeae* Filice, 1952, *G. cricetidarum* n. sp. *Lyu 2018*, *G. muris* Filice, 1952, *G. microti* Filice, 1952, *G. peramelis* Hillman, 2016, *G. psittaci* Filice, 1952, and *G. duodenalis,* Styles, 1902 (syn. *Cercomonas intestinalis* Lambl, 1859; *Lamblia intestinalis* Blanchard, 1888; *G. lamblia* Kafoid and Christiansen, 1915)—that can infect wide range of mammals, including humans [1]. *G. duodenalis* is the only protozoan zoonotic species which is grouped in eight assemblages (A–H): A and B are infectious to humans and other mammals; E is infectious to livestock, including cattle, but non-zoonotic; the rest of assemblages are non-zoonotic and may infect different host species [2].

Giardiasis is *G. duodenalis*-caused infection in cattle with enteric manifestations accompanied with diarrhoea. The severity of clinical signs are age-dependent, with symptoms being more prominent in calves younger than 30 days old. Clinically, giardiasis may be observed in cattle until they are six months of age [3,4,5,6]. After exposure of calves to *G. duodenalis* infection, gastric acid disrupts the cyst wall with initiation of excystation after a short excyzoite stage. Later, *Giardia* turns into vegetative stage trophozoite, which colonizes the epithelium of the small intestine through attachment with an adhesive disc and multiplies by longitudinal binary fission every 6–12 h [7]. Giardiasis may develop as malabsorption induced by reduced jejunum brush border surface area and decreased absorption of fluids, electrolytes, and nutrients. These conditions may lead to increased intestinal motility, reduced production, and activity of intestinal enzymes, including disaccharidase, maltase, and lactase, hence decreased weight gain [6,8,9].

Giardiasis causes immense economic losses, especially in meat- and milk-oriented cattle farms, attributed to high treatment costs, calf mortality, and decreased weight gain in animals [10].

*Giardia* has been found in beef and dairy cattle worldwide [11]. Prevalence can vary between farms with different management systems, such as differences in animal husbandry and production strategies. Geographic areas and climate conditions have also been associated with differences in *Giardia* prevalence in studies [4]. Study design may also affect the reported prevalence, and longitudinal epidemiological studies have consistently demonstrated prevalence of up to 100% in beef and dairy cattle herds [8,9]. Transmission of *Giardia* occurs between infected and non-infected calves, but chronically infected, asymptomatic adults may also be involved. The increased cyst excretion in dams has been demonstrated during the periparturient period that involves the period between two weeks before calving and four weeks post-calving; thereby, dams can also infect calves [12]. Infected calves can start to excrete the cysts as early as four days after the animal has been infected. Typically, the maximum number of colonizing cysts is reached within five weeks after infection, and infected animals can shed the cysts for several months [4,6,13]. *Giardia* transmission occurs between calves, despite the use of extensive disinfection and antiparasitic treatment [8]. Broad-spectrum anthelmintic drugs—benzimidazoles (albendazole, fenbendazole) group and paromomycin—are used for the treatment of giardiasis, and antiparasitic therapy has been shown to reduce cyst excretion and length of diarrhoea [8,14]. Although there are no licensed drug treatments for giardiasis for ruminants in Europe, benzimidazoles are relatively safe to use, if used appropriately and in combination with proper control to prevent reinfection, such as heat, disinfection with 10% ammonia, and desiccation. *Giardia* cysts are resistant to chlorine and may survive in that environment [14].

Calves should be considered a major source of environmental contamination since the infected animal may excrete as much as 10^6^ cysts per gram of faeces [14]. The infective dose of *G. duodenalis* can be as low as ten cysts [15]. Cysts are infectious immediately after excretion and can survive for a long period in the environment: in water, soil, and faeces at +4 °C up to nine weeks; and in cattle slurry for three months [16,17,18,19]. Although the prevalence of *G. duodenalis* in dairy and beef cattle farms in Europe is extensively studied, there is a necessity to recognize the risk factors associated with the carriage of *G. duodenalis* on the farm. The systematic review aims to estimate the prevalence of *G. duodenalis* and its assemblages in cattle to identify the most important risk factors for cattle giardiasis in Europe.

## 2. Materials and Methods

### 2.1. Search Strategy and Criteria Selection

A systematic review was performed to collect information on the prevalence of *G. duodenalis* in cattle; the presence of *G. duodenalis* assemblages in cattle; and the most important risk factors related to the prevalence of giardia in cattle in Europe. International online databases PubMed, Web of Science, ScienceDirect (all fields), and Scopus (title, abstract, and keywords) were searched for all published data on the topic. The “PRISMA” “Preferred reporting items for systematic reviews and meta-analyses (PRISMA)” guidelines were followed (Supplementary file S1) [20]. The following search phrases were used: Giardia (AND) Cattle (AND) Prevalence (AND) Risk factors. The databases were searched for all published studies in English, from 1960 to 1 November 2022.

### 2.2. Eligibility Criteria, Study Selection, and Data Extraction

The search results from four databases were combined and duplicates were excluded. Eligible studies were initially selected based on the title and abstract using the inclusion and exclusion criteria. Inclusion criteria were: study was done on cattle and contained information about the prevalence of *G. duodenalis*; study was done in European countries (European Union, European Economic Area, and the United Kingdom); and study was published from 1960 to 1 November 2022. Exclusion criteria were: studies of *Giardia* prevalence in animal species other than cattle, humans, or environment; studies done outside Europe; letters, editorials, notes, books, comments, case reports, reviews, and conference abstracts. Primary screening of the titles and abstracts of each study was conducted to judge the eligibility for the present systemic review. Additionally, the reason(s) for exclusion during the primary screening was recorded. If two reviewers could not reach an agreement on inclusion or exclusion after the primary screening, the titles and abstracts were screened for eligibility by a third independent reviewer till a consensus was reached. Studies were further screened for eligibility based on the full text by two independent experts. Exclusion criteria for the second screening were: studies with no full text available; conference abstracts; and no prevalence data included. The data representing the eligible studies for the full-text screening were collected and processed in Microsoft Excel 365 spreadsheets. The following variables were extracted from each study: country, study year, number of farms visited, collected samples, positive samples, prevalence, cattle age, sample preparation technique, diagnostic tools, diagnostic kit for cyst detection, DNA isolation technique, PCR type, targeted gene, assemblage, and on-farm risk factors (Supplementary file S2).

### 2.3. Statistical Analysis

The total prevalence and the pooled prevalence were calculated using the open-source software for epidemiological statistics OpenEpi (OpenEpi: Open Source Epidemiologic Statistics for Public Health, Version. www.OpenEpi.com, accessed on 2 November 2022) [21] by calculating 95% confidence interval using the Z-test for estimation of differences between the population. To determine significances of all statistical analyses, *p*-value of 0.05 was used. Fisher’s exact test (*p* < 0.05) was used to calculate the differences in *Giardia* prevalence between cattle of different age groups.

## 3. Results

### 3.1. Search Results and Eligible Studies

**Figure 1:**
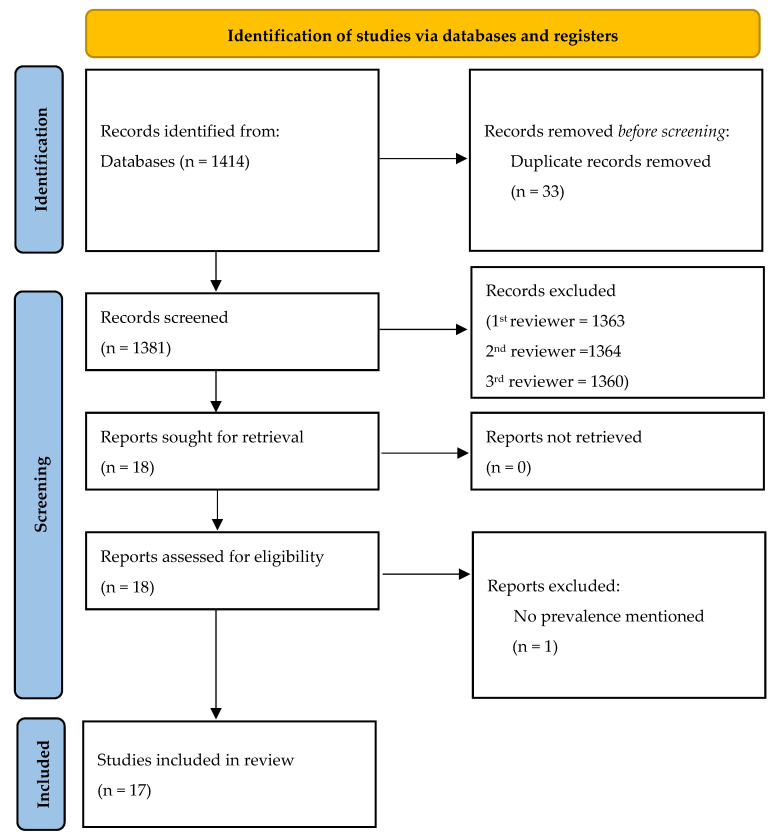
Flow diagram of the included studies.

In the literature search, a total of 1414 studies were identified (Figure 1). Among the screened papers, 33 studies were duplicates and 1364 studies did not meet the inclusion criteria during either the title and abstract, or the full-text screening. Altogether, 17 studies were found relevant for the present review from following countries—Belgium, Denmark, France, Germany, Italy, Norway, Scotland, Spain, The Netherlands, and the United Kingdom (one from 1996, nine from 2000 to 2010, and seven from 2011 to 2020)—after thorough screening for the inclusion and exclusion criteria (Table 1). 

### 3.2. Study Design

In all studies, there was no unified age distribution for cattle, therefore for pooled prevalence calculations, age groups were divided as follows: neonatal (≤1 month old), calves (1–6 months old), heifers (7–24 months old), and adults (≥24 months old). If the included study aimed to detect the prevalence of giardia within the farm independently of animal age, the prevalence was shown as the total prevalence (Table 2). 

The number of sampled farms was mentioned in 14 studies (mean 46.7, 95% CI: 31.4–78.9) and collected sample size was mentioned in 14 studies (mean 334.7, 95% CI: 323.6–345.7).

Sample collection per rectum was mentioned in thirteen studies, but the sampling technique was not mentioned in four studies.

### 3.3. Giardia Duodenalis Detection in Faeces

#### 3.3.1. Cyst Detection

Microscopic methods were used for cyst detection in 13 studies. Five different protocols were used for faecal sample preparation for microscopy: a purification technique with saturated saline and glucose flotation fluid was used in two studies, a flotation technique with sucrose fluid, a formalin-ethyl acetate sedimentation technique, a Merthiolate-iodine-formaldehyde concentration, and a sodium acetate–acetic acid–formaldehyde technique were used in one study each, and no sample preparation was used in seven studies.

Two different staining protocols for cyst detection in faecal samples were used in nine studies: an immunofluorescence staining technique (IMF) (n = 8, 90.9%, 95% CI: 60.1–100.0) and iodine staining (n = 1, 9.1%, 95% CI: 0.0–39.9). For IMF, three different kits were used: Aqua-Glo G/C FL (Waterborne Inc., New Orleans, LA, USA) (n = 4, 50.0%, 95% CI: 21.5–78.5%), *Crypto/Giardia*-Cel FITC Stain (CelLabs, Brookvale, Australia), and MERIFLUOR *Cryptosporidium/Giardia* (Meridian Diagnostics Inc., Cincinnati, OH, USA) (n = 2 each, 25.0%, 95% CI: 6.3–59.9). In two studies, no microscopic cyst detection was performed.

#### 3.3.2. Indirect Detection Methods

In three studies, indirect detection methods for giardia detection were used. The enzyme-linked immunosorbent assay (ELISA) was applied in two studies with CpAG-ELISA (*Giardia lamblia* antigen-ELISA, Novatec Immunodiagnostica GMMH, Dietzenbach, Germany) and GIARDIA II™ (Techlab, Blacksburg, VA, USA). The immunochromatography (ICT) assay with Stick *Crypto-Giardia* (Operon S.A., Zaragoza, Spain) was used in one study.

#### 3.3.3. Molecular Methods

Species and assemblages were detected with molecular analyses in 11 studies.

DNA was directly isolated from faeces in eight studies (72.7%, 95% CI: 42.9–90.79) and from purified samples in two studies (18.2%, 95% CI: 4.0–48.5). Concentration of a sample with Merthiolate iodine formaldehyde (9.1%, 95% CI: 0.0–39.9) was conducted in one study.

DNA isolation was done with four different isolation kits: the QIAamp DNA stool mini kit (QIAGEN GmbH, Hilden, Germany) (n = 5, 45.4%, 95% CI: 21.2–72.0) was the most used, followed by the QIAamp DNA mini kit (Qiagen, Valencia, CA, USA) (n = 2, 18.2%, 95% CI: 4.0–48.5), the Maxwell 16 tissue DNA purification kit (Promega, Madison, WI, USA) and the NucleoSpin Tissue DNA columns (Macherey-Nagel GmbH & Co., Düren, Germany) (n = 1 each, 9.1%, 95% CI: 0.0–39.9). DNA isolation protocol was not mentioned in two studies (18.2%, 95% CI: 4.0–48.5).

For giardia detection, the β-giardin (*bg*) gene was targeted in seven studies (87.5%, 95% CI: 50.8–99.9), and the 292-bp fragment of the small subunit ribosomal (SSU) rRNA (18S rDNS) in one (12.5%, 95% CI: 0.1–49.2) study. In nine studies, the glutamate dehydrogenase (GHD) or triosephosphate isomerase (TPI) genes were targeted simultaneously or separately for *Giardia* assemblage detection. Only the GHD gene was targeted (44.4%, 95% CI: 18.8–73.4) in four, while only the TPI gene (11.2%, 95% CI: 0.0–45.7) was targeted in one study, and both genes were screened in four studies (44.4%, 95% CI: 18.8–73.4). In seven studies, multiple genes were targeted simultaneously: either *bg*, TPI, and GHD genes (n = 3, 42.8%, 95% CI: 15.7–75.0), β-giardin and GHD (n = 2, 28.6%, 95% CI: 7.5–64.8), TPI and GHD, or *bg* and TPI (n = 1, 14.3%, 95% CI: 0.5–53.4). 

In total, three different PCR methods were used: conventional PCR (n = 4, 40.0%, 95% CI: 16.7–68.8), nested-PCR (n = 4, 40.0%, 95% CI: 16.7–68.8), and semi-nested PCR (n = 2, 20.0%, 95% CI: 4.6–52.1). In one study, the PCR method was not mentioned.

### 3.4. Prevalence of G. duodenalis in Europe

*Giardia* prevalence in cattle in Europe was 35.1% (95% CI: 34.3–35.9), with the pooled mean prevalence in neonatal calves of 39.6% (95% CI: 36.4–42.8), calves of 38.2% (95% CI: 35.1–41.3), heifers of 10.7% (95% CI: 8.5–13.2), and adults of 14.2% (95% CI: 12.6–15.9) (Table 2). In neonatal animals, a significantly higher prevalence of *Giardia* than in other age groups (*p* < 0.05) was found. 

The mean number of cysts per one gram of faeces for animals of all ages was 9737.2 (95% CI: 9560.6–9913.8), ranging from 1 to 94,000. The mean farm prevalence was 67.0% (95% CI: 56.2–78.0), ranging from 42.9% to 96.9%. 

Assemblages were successfully differentiated in nine studies. Mixed infections of A and E assemblages were found in five studies (55.6%, 95% CI: 26.6–81.2), only assemblage E was found in three studies (33.3%, 95% CI: 11.7–64.9), and mixed infection with A, B, and E assemblages was found in one study (11.1%, 95% CI: 0.0–45.7). In one study, no data about assemblages were obtained because of failure in DNA isolation. Assemblages A and B were found in cattle younger than 24 months.

### 3.5. Risk Factors of G. duodenalis in Cattle Farms

#### 3.5.1. Farm-Related Factors

Farm-related factors were analysed in six studies. Factors—herd size, organic or conventional farm, cattle breed, cattle kept outdoors or indoors, and floor type (slatted or solid)—did not show an impact on *G. duodenalis* prevalence and cyst excretion intensity [23,25,30,38]. Higher *G. duodenalis* prevalence in dairy cattle compared to beef cattle was observed in one study [31]. Significantly higher prevalence of *Giardia* was observed in German Angus calves kept on deep litter with run-out compared to deep litter housing without run-out, winter run-out yarding, or slatted floor management systems [26]. The regular cleaning of maternity pens (4x/year), instead of cleaning them only after calving, reduced the *Giardia* infection risk in calves [25].

#### 3.5.2. Calf Husbandry-Related Factors

In three studies, factors related to calf rearing practice were discussed. Individual or group housing of calves did not reveal significant impact on the presence of *Giardia*, while a higher risk was identified in calves after contact with the dam [25]. The disinfection of housing units significantly reduced giardiasis in calves [25]. Maddox-Hyttel et al. [23] found that excretion of *Giardia* cysts was significantly lower in farms which had implemented high-pressure cleaning and maintained the empty period prior to the introduction of new calves into a pen. A significantly lower prevalence was observed in individually fed calves older than three weeks as compared to group-fed calves (*p* = 0.0030) [30].

#### 3.5.3. Seasonality

In three studies, the differences in the prevalence of *G. duodenalis* between seasons were observed. In The Netherlands and Norway, the prevalence of *Giardia* in cattle was higher in winter than in summer or autumn, respectively [30,37]. In contrast, in Spain, prevalence was significantly higher in spring (*p* < 0.05) than in summer and winter [36].

#### 3.5.4. Age

In five studies, animal age was identified as a risk factor of for *Giardia* infection. Higher risk was observed in calves younger than eight weeks of age [23], calves under one month of age [23,35], pre-weaned [31], suckling, and weaning calves [33].

#### 3.5.5. Presence of Diarrhoea

In five studies, the correlation between diarrhoea and *Giardia* infection was calculated. However, links between *G. duodenalis* infection, diarrhoea, and changes in faecal consistency were not established [23,25,26,33,38]. No other clinical signs were studied.

## 4. Discussion

The limited number of published studies shows that *Giardia* is common but neglected in cattle in Europe, despite its relevance as a food and waterborne parasite [15,39]. The overall pooled prevalence in Europe reached 35.1%.

Different sample preparation techniques were used for the microscopic analysis of animal faeces. Staining with iodine is a fast, cheap, and easy to perform the diagnosis of *Giardia*; however, lower sensitivity and specificity compared to IMF has been reported [40]. Indirect detection methods possess higher sensitivity than iodine staining or light microscopy as opposed to IMF, but may lead to incorrect results, if a low number of cysts is present. Indirect detection methods were reported to be more expensive than IMF, which might affect their availability and practical application [41]. Despite the IMF being “the gold standard” for the detection of *Giardia* cysts in faeces, expensive equipment and trained staff are required for testing. In contrast, ELISA is an easy to perform, cost-effective, and commercially widely available protocol for the routine diagnosis of giardia. Additionally, only minor differences between the sensitivity and specificity of direct immunofluorescence and ELISA methods were found, which demonstrates the practical importance of both laboratory investigations [42]. The classical detection methods include the microscopic detection of *Giardia* cysts by flotation and iodine staining; however, false negative results which arise due to the application of those methods have been reported [43].

Direct DNA isolation from faeces was done in most of the studies. Better results from the application of DNA isolation were attributable to the additional cleaning of debris, aiming to remove large amounts of bacteria and other DNA present in the faeces, which is also less time consuming than purifying [44]. The β-giardin gene is unique to *Giardia*, and is therefore frequently targeted for the detection of the pathogen. Additional advantages include the absence of cross-reaction with other pathogens, such as fungi or bacteria as compared to 18S rRNA gene which lacks specificity, and the 18S rRNA gene-amplified specimens were negative for other genes (*bg*, TPI, and GHD) [38,44,45]. Even though a single gene can be targeted for the detection of *Giardia*, simultaneous identification of all three *Giardia* genes is required for genotyping and subtyping [18]. Conventional PCR was mostly used, even though semi-nested and nested PCR can retrieve better results if low numbers of cysts are present in the sample [46]. Conventional PCR is the gold standard for the routine diagnosis of *Giardia* because of the lower costs and wider availability, even though there is a need for experienced technical staff [47,48].

Results on the prevalence of *Giardia* between age groups differed, but the highest prevalence was found in neonatal animals. Overall, the highest prevalence was recorded in calves younger than one month [23,31,37] or in calves between one and six months of age [33,39]. Observed differences in the prevalence of *Giardia* between the cattle age groups could be related to prolonged periods of cyst excystation, which may extend to 112 days after initial infection [6,8]. Assemblage E has been found in all cattle age groups; however, an association between the presence of this assemblage and the clinical diarrhoea was not identified [48,49,50]. The prevalence of assemblages A and B in calves is of importance since both share zoonotic potential and expressed pathogenicity in humans [50,51,52]. Similar findings were reported from South Korea, the USA, and Canada, where the highest prevalence was observed in calves 7–8 and ≥12 weeks (15.4%) old, calves 2–11 months (52.0%) old, and calves under 6 months old (53.0%), with zoonotic assemblage A (3.8%; 12.2%; 13.0%, respectively) presence [53,54,55].

In several studies, the risk analysis has been conducted to identify the factors affecting the distribution of *Giardia* between farms. It has been observed that beef cattle herds had higher prevalence compared with dairy cattle, which could be due to different management systems [22,31,54]. Even though multiple studies recorded from which dairy or beef cattle breed samples were collected [22,25,56], information about potential prevalence differences between specific breeds is lacking.

Deep litter housing without run-out, use of straw bedding, and slatted floor types were associated with a potential risk of excretion and accumulation of cysts in the environment [23,26]. Incorrect use of deep litter housing and the use of straw bedding can retain moisture that creates appropriate microclimatic conditions for parasite survival [23,57]. Prolonged dam–calf contact or contact with other calves increased the odds of infection, because of the potential rise in cyst excretion in the dams’ peri-parturient period and the large accumulation of cysts in the environment over time from other infected calves were observed in France, Italy, Germany, and the United Kingdom, as well as the USA [25,58]. Regular cleaning of maternity pens reduced the shedding of *Giardia*, and routine high-pressure cleaning of pens with disinfection and an empty period before the housing of calves reduced cyst excretion in calves [23,25]. Similar results were observed in the USA [59]. These farm management-related risk factors show that proper prevention and improvement in animal farming practice could significantly reduce the prevalence of acute or chronic giardiasis in cattle farms, thereby reducing the risk of economic losses. Reduced environmental contamination that may arise from the minimized emission of *Giardia* from cattle farms should be considered a potential benefit of improved animal farming strategy.

Higher prevalence of *Giardia* was observed in winter and spring, rather than summer or autumn, months. A higher prevalence of giardia in cattle in winter months could be linked to lower immunity, higher density of animals indoors, and prolonged survival of cysts in colder temperatures. Additionally, widespread occurrence of respiratory infections in colder months may provoke the development of giardiasis as a secondary infection [30,37,60]. 

Age as a risk factor was discussed in several studies, with calves under eight weeks of age being more affected. Newborn calves are protected with the dam’s colostrum, that contains immune cells and may contain anti-giardia antibodies, providing passive immunity against giardia with the maximum protective effect lasting in calves up to two weeks of age [8,13].

Diarrhoea was observed in cattle, but associations between diarrhoea and infection were not found, apart from one study done by Xiao et al. [61]. Diarrhoea in infected calves could be induced by other enteric pathogens, such as *Cryptosporidium* or rotavirus. *Giardia* may cause chronic, intermittent diarrhoea that makes it difficult to establish a connection between the clinical signs and the presence of the pathogen [23,25,26,33,37,62]. 

Even though sex as a risk factor was not included in these studies, there are studies done in Turkey and South Korea that show no differences in prevalence between males and females [53,63]; however, other studies have shown that prevalence was higher in male cattle in Nepal and Bangladesh, which could be due to different housing conditions for male calves [64,65,66].

## 5. Conclusions

This review provides a limited, but comprehensive overview of the presence of *Giardia* in cattle in Europe. The prevalence of giardia was significantly different between the animal groups, with neonatal cattle being more clinically affected, while older cattle could be potential asymptomatic carriers. Assemblages A and B were identified in cattle of all ages that could indicate a potential zoonotic threat for humans. Application of DNA-based methods may improve the sensitivity and specificity of the detection of *Giardia* infection in animals. The estimation of potential risk factors may be helpful in establishing pathogen on-farm control programmes for the reduction of giardia in cattle.

## Figures and Tables

**Table 1:** Characteristics of 17 eligible studies on *Giardia duodenalis* in cattle in Europe.

Country	Study Year/-s	Microscopic Cyst Detection	PCR Confirmation	Assemblage Detected	Reference
Belgium	2001–2005	IMF	Yes	Yes	[22]
Denmark	2003–2005	IMF	No	N/A	[23]
Denmark	2003–2004	IMF	Yes	Yes	[24]
France	2010	ELISA ^b^	Yes	Yes	[25]
Germany	n.r.	Light microscopy	No	N/A	[26]
Germany	2011–2013	Direct smear	Yes	Yes	[27]
Germany	2010	ELISA ^b^	Yes	Yes	[25]
Greece	2017	IMF	Yes	Yes	[28]
Italy	2010	ELISA ^b^	Yes	Yes	[25]
Norway	2004–2007	IMF	No	N/A	[29]
Norway	2001–2003	IMF	No	N/A	[30]
Scotland	n.r.	Not done	Yes	Yes	[31]
Spain	n.r.	IMF	Yes	Yes	[32]
Spain	1990–1993	Iodine staining	No	N/A	[33]
Spain	2008–2010	IMF	Yes	Yes	[34]
Spain	2008–2009	ELISA ^a^, ICT	Yes	No	[35]
Spain	2007	IMF	No	N/A	[36]
The Netherlands	1999–2000	IMF	No	N/A	[37]
United Kingdom	2007–2008	Not done	Yes	Yes	[38]
United Kingdom	2010	ELISA ^b^	Yes	Yes	[25]

IMF—immunofluorescence staining; N/A—not appliable; ^a^ Coproantigen ELISA (*Giardia lamblia* antigen-ELISA, Novatec Immunodiagnostica GMMH, Dietzenbach, Germany); ^b^ monoclonal antibody-based ELISA (Giardia II, catalogue No. 30405, Techlab, Blacksburg, VA, USA); n.r.—not reported.

**Table 2:** Summary of estimated pooled *Giardia duodenalis* prevalence in cattle by age groups (neonatal (≤ 1 month old), calves (1–6 months old), heifers (7–24 months old), adults ≥24 months old) and total prevalence of all cattle.

Country	No. of Studies Included	Age Group	No. Positive Animals	Total No. of Animals	Pooled Prevalence, % (95% CI)	Assemblage	References
Belgium	1	Total	139	832	16.7 (14.3–19.4)	A, E	[22]
Denmark	1	Neonatal	229	377	60.7 (55.7–65.5)	N/A *	[23]
		Calves	221	518	43.7 (38.5–47.0)	N/A *	
		Adults	51	255	20.0 (15.5–25.4)	N/A *	
	2	Total	1002	5300	18.9 (17.9–20.0)	E	[23,24]
France	1	Total	477	190	39.8 (35.6–44.3)	A, E	[25]
Germany	2	Total	384	688	55.8 (51.2–59.5)	A, E	[25,27]
Greece	1	Total	105	256	41.3 (35.1–47.1)	A, E	[28]
Italy	1	Total	503	162	32.2 (28.3–36.4)	A, E	[25]
Norway	2	Total	1187	2145	55.3 (53.2–57.4)	N/A *	[29,30]
Scotland	1	Neonatal	54	179	49.0 (46.4–37.3)	A, B, E	[31]
		Calves	54	167	32.3 (25.7–39.7)	B, E	
		Heifers	18	42	42.9 (27.7–59.0)	B, E	
		Total	126	388	32.5 (28.0–37.3)	A, B, E	
Spain	2	Neonatal	40	217	18.3 (13.8–25.1)	A, E	[33,36]
	1	Calves	46	121	38.0 (29.8–46.9)	N/A *	[33]
	2	Heifers	48	610	7.8 (6.0–10.3)	A, E	[33,36]
	3	Adults	155	1283	12.1 (10.4–14.0)	A, E	[32,33,36]
	5	Total	306	2645	11.6 (10.4–12.8)	A, E	[32,33,34,35,36]
The Netherlands	1	Neonatal	29	112	25.9 (18.6–34.7)	N/A *	[37]
	1	Calves	10	71	14.1 (7.6–24.2)	A	
	1	Total	39	183	21.3 (16.0–27.8)	A	
United Kingdom	1	Neonatal	7	22	31.8 (16.2–52.8)	A, E	[38]
	1	Calves	34	78	43.6 (33.1–54.6)	A, E	
	1	Heifers	6	23	26.1 (12.7–46.8)	A, E	
	1	Adults	32	139	23.0 (16.8–30.7)	A, E	
	2	Total	384	818	44.9 (43.5–50.4)	A, E	[25,38]

* N/A-not appliable.

## Data Availability

Not applicable.

## Supplementary Materials

The following supporting information can be downloaded at: https://www.mdpi.com/article/10.3390/microorganisms11020309/s1, S1: PRISMA checklist, S2: Data extracted from included studies.
